# Influence of E-Liquids and Oral Commensal Bacteria on the Growth of *Porphyromonas gingivalis* Planktonically and in Biofilms

**DOI:** 10.3390/dj14030172

**Published:** 2026-03-17

**Authors:** Sabeen Safi, Danna Berro, Juliette Amram, Daniel Burden, Dominic Palazzolo, Giancarlo A. Cuadra

**Affiliations:** 1 Biology Department, Muhlenberg College, Allentown, PA 18104, USA; ssafi@muhlenberg.edu (S.S.); dberro@muhlenberg.edu (D.B.); jamram@muhlenberg.edu (J.A.); daniel.burden@cuanschutz.edu (D.B.); 2 School of Dental Medicine, University of Colorado, Aurora, CO 80045, USA; 3 Debusk College of Osteopathic Medicine, Lincoln Memorial University, Harrogate, TN 37752, USA; dominic.palazzolo@lmunet.edu

**Keywords:** e-liquids, bacterial growth, bactericidal, biofilms, commensal bacteria, electronic cigarettes, oral cavity, streptococci, *Porphyromonas gingivalis*

## Abstract

**Background**: The increasing use of electronic cigarettes (ECIGs), especially among youth, has raised concerns about the impact of vaping on oral health. While ECIGs are often marketed as a safer alternative, the existing literature suggests that their use may have detrimental effects on the pulmonary and cardiovascular systems. The oral cavity is the first point of contact for ECIG aerosol, and new reports link vaping to the onset of periodontal disease. It is critical to understand the potential effects of vaping on the oral microbiome, which affects systemic health. This study investigates how flavored E-liquids and commensal bacteria influence the growth of *Porphyromonas gingivalis,* a periodontal pathobiont, under planktonic and biofilm conditions. **Methods**: *P. gingivalis* was grown planktonically in the presence of the supernatants of four streptococcal species (*Streptococcus gordonii*, *Streptococcus intermedius*, *Streptococcus mitis,* and *Streptococcus oralis*) and flavored E-liquids (tobacco, menthol, cinnamon, strawberry, and blueberry) under anaerobic conditions. Multispecies biofilms, including all the species mentioned above and *Fusobacterium nucleatum*, were also grown anaerobically and quantified by crystal violet assays, qPCR, and CFU counts. **Results**: Although E-liquids inhibit *P. gingivalis* growth under planktonic conditions, the presence of commensal supernatants partially mitigates this effect. However, *P. gingivalis* growth in multispecies biofilms is increased by E-liquid treatments. **Conclusions**: This study highlights the enhanced growth of *P. gingivalis* as part of an oral microbial community in the presence of E-liquids. These results suggest that E-liquid-induced alterations in multispecies biofilms may contribute to the observed dysbiosis in vapers and the associated risk of oral diseases.

## 1. Introduction

The use of electronic cigarettes (ECIGs) has become increasingly popular, especially among adolescents [1,2]. These devices were first introduced in China in 2003 as a safer alternative to smoking [3,4] and have since exploded into a worldwide public health issue [5,6]. In their simplest form, ECIGs consist of a battery, a heating coil, a removable cartridge that holds the E-liquid, and a mouthpiece [3]. E-liquids consist of vegetable glycerin, propylene glycol, various flavoring agents, and different concentrations of nicotine, which are often unregulated [7,8,9]. The coil heats the E-liquid, producing an aerosol that is inhaled similarly to traditional cigarette smoke—a process known as vaping [9].

Young adults are the primary target of ECIG marketing [3], and the myriad of palatable E-liquid flavors is used as the enticement [10]. A study including nearly four thousand participants showed that tobacco and menthol are the most popular flavors [11]. Even though many states restrict their sale to minors, young adults are able to acquire vaping products. Vaping is marketed as a healthier alternative to traditional cigarette smoking or as a means to abstain from the latter [12,13] due to its minimal ingredient list and non-combustible nature. Whether or not this is true, the existing literature shows that vaping can be harmful [9,14,15]. For example, vaping has been linked to damage to the pulmonary, cardiovascular and nervous systems [14,16,17,18]. Thus far, the flavoring components of E-liquids have been shown to cause the most detrimental effects on various models [19,20,21,22,23,24,25]. However, high concentrations of nicotine in E-liquid, the formation of volatile organic compounds from the heating of E-liquid [26,27], and the presence of trace amounts of metals leached from ECIG devices themselves [28,29] may contribute to harmful vaping-induced effects in the airway [30] and oral cavity [31]. While there is substantial research on the effects of E-liquids on the respiratory system, our understanding of their effects on the oral cavity is still developing.

Emerging clinical evidence reveals concerning patterns of oral health deterioration among ECIG users, commonly known as vapers. Studies comparing periodontal parameters among cigarette smokers, vapers, and non-smokers have shown that conventional smokers exhibit the poorest periodontal status with elevated inflammatory mediators, while vapers demonstrate intermediate and unique outcomes between smokers and non-smokers [32], as well as increases in pathobionts and suppression of commensals [33,34]. Thus, vapers have a moderate risk for periodontal health issues [15,35,36,37]. In addition to periodontal disease, vapers were more likely to have untreated dental caries compared to non-smokers [38,39,40]. Microbiome analyses also display an increase in the abundance of pathogenic bacteria, including the Gram-negative anaerobe and periodontal pathobiont *Porphyromonas gingivalis*, among vapers [38,41,42,43]. Such observational studies indicate that vaping may also be associated with an increased risk for periodontal disease, and that *P. gingivalis* plays a key role. Furthermore, it is essential to note that oral and systemic health are intimately related. For example, periodontal disease is associated with a range of systemic conditions, including diabetes and cardiovascular disease [44,45]. Therefore, vaping may have short and long-term effects on both oral and systemic health.

The oral cavity contains one of the highest concentrations of bacteria in the human body, harboring over 700 microbial species [46]. These microorganisms typically exist as biofilms, structured communities of bacteria adhered to oral surfaces, which include live organisms, dead cells, microbial metabolites, an exopolysaccharide layer that covers the community (slime layer), extracellular DNA, and nutrients that flow in and out of the biofilm [47]. Oral biofilms live symbiotically with the host and play a central role in homeostasis and oral health [48,49]. Pathobionts reside in these complex microbial communities, and the balance between health and disease depends on the multispecies interactions with the host [50]. These interactions include symbiotic and antagonistic mechanisms within oral biofilms. For example, *P. gingivalis* can coexist with commensals in a mutualistic balance as commensals remove oxygen, enabling *P. gingivalis* to thrive [51]. Oral commensal *Streptococcus gordonii,* an early colonizer of oral surfaces, produces ornithine, which is further metabolized by another Gram-negative anaerobe, *Fusobacterium nucleatum,* generating polyamines and putrescine, and these accelerate maturation and dispersal of *P. gingivalis* [52]. In symbiosis with the host, *S. gordonii* produces arginine deiminase, which metabolizes arginine to generate ammonia, thereby neutralizing acids in dental plaque and protecting tooth enamel from demineralization [53]. In contrast, many commensal streptococci help maintain microbial balance on oral surfaces by producing hydrogen peroxide, minimizing the overgrowth of pathobionts, and thereby helping maintain homeostasis [54]. A prior study conducted by our team showed that the oral commensals *S. gordonii* and *Streptococcus intermedius* defend against invasion of *P. gingivalis* into oral epithelial cells [55], exemplifying that oral homeostasis is dependent upon interactions, both symbiotic and antagonistic, between microbial species, leading to a balance for both the microbes and the host.

Oral bacteria exist as planktonic (free-floating) cells in saliva and in gingival crevicular fluid as well as surface-attached (sessile) biofilms on tooth enamel and the periodontium. In periodontal disease, the clinically relevant form is the biofilm state, where bacteria exhibit altered gene expression and enhanced resistance to antimicrobials, and exacerbate inflammatory responses that degrade underlying oral tissues [48,56,57]. The existing literature on E-liquid effects on the oral microbiome often focuses on planktonic bacterial cultures, with fewer studies examining biofilm responses. Furthermore, E-liquid toxicity may be mitigated by metabolic cross-feeding, as streptococcal metabolites can support *P. gingivalis* survival under anoxic conditions [58,59,60] and, as observed in other bacterial systems, metabolic defense mechanisms can reduce oxidative stress [61]. Consequently, any pathobiont in a multispecies environment could benefit from the presence of other microbes and/or their metabolic derivatives. Moreover, the two growth states exhibit different physiological and metabolic characteristics [62,63,64,65,66]. Assessing E-liquid effects on both states is therefore essential to understanding *P. gingivalis* behavior in vivo since this pathobiont exists in both planktonic and sessile states surrounded by other microorganisms, including commensals.

Studies by our group have shown that traditional cigarette smoke is more harmful to oral commensal streptococci compared to flavorless E-liquid or its aerosol [67,68], albeit the effects of flavored E-liquids, particularly menthol and cinnamon, on oral commensals show dose-dependency. Historically, our lab has shown that at lower E-liquid concentrations (<1%), commensal bacteria are not adversely affected; in fact, some strains exhibit improved growth under these conditions [69,70]. At higher E-liquid concentrations (up to 5%), a clear dose-dependent reduction in biofilm formation is evident with all flavors, but mainly menthol and cinnamon, which also induce cell death [69,70,71]. These findings indicate that E-liquids can directly destabilize oral homeostasis. Since in vivo evidence suggest that there is a positive correlation between *P. gingivalis* growth and vaping [33,34] and that oral biofilms in vitro are altered by E-liquids, it is possible that this pathobiont can thrive in biofilms exposed to E-liquids in vitro.

Given the increased abundance of *P. gingivalis* in vapers’ subgingival plaque and its role as a keystone periodontal pathobiont [72], this study aims to develop an in vitro model designed to mimic, at least in part, the effects of E-liquids on (i) the planktonic growth of *P. gingivalis* alone and in the presence of *S. gordonii*, *S. mitis*, *S. oralis*, and *S. intermedius* supernatants, individually or pooled together, and (ii) the growth of *P. gingivalis* in multispecies biofilms. Our six-species model includes the four oral streptococci [73] as early colonizers that establish the biofilm foundation, *F. nucleatum* as a bridging organism that facilitates attachment of late colonizers [74], and *P. gingivalis* as the target periodontal pathobiont. While this approach cannot capture the full complexity of natural oral biofilms, it enables controlled examination of interspecies interactions at the most fundamental level. We hypothesize that E-liquids ± flavors inhibit *P. gingivalis* growth, but streptococci or their by-products present in the supernatant may mitigate this toxic effect either planktonically or in multispecies biofilms. Understanding these effects may help clarify how vaping influences *P. gingivalis* within oral microbial communities, which could lead to periodontal disease.

## 2. Materials and Methods

### 2.1. Study Design

All experiments were performed in the Cuadra laboratory at Muhlenberg College. E-liquids and components were originally designed in the Palazzolo laboratory at Lincoln Memorial University. Based on our previous studies of bacterial cultures and those of others, we find that typically n = 10 is sufficient to perform statistical analyses. For all experiments, two to six independent experiments were performed and each experiment included three to six biological replicates; the total theoretical n value ranges from 6 to 36 (actual n values range from 4 to 18). For all molecular analyses (qPCR), every biological replicate only included a single technical replicate. Every biological replicate equates to a single technical replicate. For all experiments, data was collected at either 0, 6, 12, 24 and 30 h or just at 24 h post-treatment. All saliva was collected with the approval of the IRB Ethics committee at Muhlenberg College (Cuadra_S19_18). Reagents and supplies for this study were purchased from Fisher Scientific (Waltham, MA, USA) unless otherwise explicitly noted.

### 2.2. Stock E-Liquids

The base flavorless E-liquid solution was prepared by mixing humectants, propylene glycol and glycerol, in a 1:1 *v*/*v* ratio (Liquid Nicotine Wholesalers, Phoenix, AZ, USA), then spiking the mixture with 20 mg/mL of (S)-(-)-nicotine, 99% (Alpha Aesar, Tewksbury, MA, USA). Stock flavors, including tobacco, cinnamon, strawberry, blueberry (Liquid Nicotine Wholesalers, Phoenix, AZ, USA), and menthol (Vapor Vapes, Sand City, CA, USA), were added to the base flavorless E-liquid at a final concentration of 5% (*v*/*v*) as outlined in our previous protocols [69,70,71]. E-liquids were stored at 4 °C after preparation.

### 2.3. Saliva Preparation

Saliva samples were collected from a minimum of five healthy donors with IRB approval (Cuadra_S19_18). As previously described [69,71], the inclusion criteria and pre-collection instructions to donors were as follows: (i) non-smokers and non-vapers, (ii) in good health at the time of donation, (iii) no antibiotic use within the three months preceding donation, and (iv) no consumption of food or beverages other than water within two hours prior to donation. No demographic data was collected from any saliva donors. Donors chewed parafilm as a stimulant for salivation and placed saliva in sterile plastic tubes on ice. Donations ranged between ≈35 and ≈45 mL per donor over a period of ≈20 to ≈30 min. Raw saliva samples were stored at −20 °C until processing. All saliva from at least five donors was thawed and pooled on ice to reduce donor-to-donor variability. To break disulfide bonds, dithiothreitol was added to a final concentration of 2.5 mM, and samples were gently stirred on ice for 10–15 min. The saliva was subsequently centrifuged at 4500× *g* for 90 min, after which the supernatant was collected and diluted 1:4 (*v*/*v*) with distilled water. The diluted saliva was filter-sterilized using a 0.45 µm vacuum filtration system. All saliva was used for the purpose of creating an acquired pellicle on all styrene (clear plastic) vessels used for biofilm formation and growth. Sterile saliva was stored at −20 °C for up to one year or at 4 °C for up to two weeks prior to use.

### 2.4. Bacterial Strains and Growth Conditions

All bacterial stocks were stored at −80 °C, and the purity of each strain was routinely verified by 16S rRNA gene sequencing (Genewiz, South Plainfield, NJ, USA) to confirm species identity.

Facultative oral streptococci used in this study included *S. gordonii* DL1, *S. intermedius* 0809, *Streptococcus mitis* UF2, and *Streptococcus oralis* SK139. These strains were kindly provided by Dr. Robert Burne from the University of Florida, College of Dentistry in Gainesville, Florida, USA. In addition, anaerobic strains *Porphyromonas gingivalis* W83 and *Fusobacterium nucleatum* ATCC 49526 were provided by Dr. Progulske-Fox and Dr. Kesavalu, respectively, at the University of Florida College of Dentistry in Gainesville, FL, USA.

Oral streptococci were cultured in brain heart infusion (BHI) agar or BHI broth supplemented with 5 μg/mL porcine hemin at 37 °C with 5% CO_2_, as described previously [30,33,43,44]. Anaerobes *F. nucleatum* and *P. gingivalis* were grown in Tryptic Soy Agar (TSA) supplemented with 5% sheep’s blood and 1 μg/mL menadione and in Tryptic Soy Broth (TSB) supplemented with 1 mg/mL yeast extract (TSBY), 1 μg/mL menadione and 5 μg/mL porcine hemin [55]. *P. gingivalis* strain W83 is resistant to 30 μg/mL gentamicin and was routinely grown in TSBY and blood agar with this antibiotic. Anaerobes were cultured in a BACTRON Anaerobic Chamber (Sheldon Manufacturing, Inc., Cornelius, OR, USA) at 37 °C, 90% nitrogen, 5% hydrogen, and 5% CO_2_.

### 2.5. Preparation of Commensal Supernatants

*S. gordonii*, *S. intermedius, S. mitis*, and *S. oralis* cultures were grown in BHI at 37 °C, 5% CO_2_, to the late exponential phase depending on microbial species. Supernatants were adjusted to physiological pH (7.0–7.8) using incremental additions of 1 M NaOH. Cultures were then centrifuged at 15,000× *g* for 10 min, and clarified supernatants were filter-sterilized using 0.45 μm filters. Filtered supernatants were aliquoted, labeled, and stored at −80 °C until use in subsequent assays.

### 2.6. Growth Curves of P. gingivalis with E-Liquids and Oral Commensal Supernatants

#### 2.6.1. *P. gingivalis* Planktonic Growth

Treatments were prepared by diluting E-liquids or their components (see Section 2.2) to a final concentration of 2% (*v*/*v*) in TSBY. Commensal supernatants (see Section 2.5) were diluted to 20% (*v*/*v*) in TSBY. Combined treatments included both 2% (*v*/*v*) E-liquid and 20% (*v*/*v*) commensal supernatants in TSBY. All treatment and control media (TSBY alone) were added to 96-well plates at 100 µL per well. *P. gingivalis* W83 was grown anaerobically on blood agar for two days. *P. gingivalis* colonies were resuspended in TSBY and adjusted to an absorbance of 0.5 at 595 nm. Then, 100 µL of *P. gingivalis* suspension was added to the above treatment (and control) media in 96-well plates, yielding a final volume of 200 µL per well. With this, the bacteria’s absorbance was diluted to roughly 0.25, and the treatments were diluted to 1% E-liquids and 10% commensal supernatants. Six wells were used to measure 200 µL of blank media to subtract media absorbance. Cultures were incubated anaerobically at 37 °C, and growth was monitored by measuring absorbance at 595 nm at defined time points up to 30 h using a µQuant monochromatic microplate reader (MTX Lab Systems, Bradenton, FL, USA) equipped with the Gen5 version 1.1 software (BioTek, Winooski, VT, USA). To ensure continuous anaerobic growth of P. gingivalis, its growth was measured at each time point using a separate 96-well plate that was read once and not returned to the anaerobic incubator. To remove background noise, TSBY OD values were subtracted from culture OD values.

#### 2.6.2. *P. gingivalis* CFU Quantification at 24 h of Planktonic Growth

At 24 h of growth (see Section 2.6.1), bacterial viability was additionally assessed by colony-forming unit (CFU) counting. Cultures were serially diluted 1:10 up to 1:10^7^ in phosphate-buffered saline (PBS), and 10 µL of the 1:10^5^, 1:10^6^, and 1:10^7^ dilutions was spot-plated in triplicate onto blood agar and incubated anaerobically for two days. Colonies were counted using a dissecting microscope.

### 2.7. Quantification of Multispecies Biofilms Exposed to E-Liquids

#### 2.7.1. Crystal Violet

Salivary pellicles were formed by coating acetone-treated sterile 96-well plates with 100 µL of processed human saliva per well and incubating overnight at 4 °C. Multispecies streptococcal biofilms were formed as previously described [33,43] with a few adjustments. Briefly, *S. gordonii*, *S. intermedius*, *S. mitis*, and *S. oralis* were grown overnight (see Section 2.4) and adjusted to the same absorbance of 0.8. The four strains were then mixed at a 1:1:1:1 ratio, yielding a final volume of 12 mL (3 mL for each species). Saliva-coated wells were inoculated with 100 µL of the bacteria mixture and incubated for 1 h at 37 °C, 5% CO_2_, to allow adherence to the surface. Non-adherent bacteria were removed by washing wells three times with 100 µL sterile PBS. Then, 100 µL of 50% TSBY was added to all wells, and the plate was incubated anaerobically for 24 h. *F. nucleatum* and *P. gingivalis* were grown anaerobically in TSBY, adjusted to the same absorbance (0.8), and combined at a 1:1 ratio. The pre-established streptococcal biofilms were washed twice with sterile PBS as above, and 100 µL of the anaerobe mixture was added to each biofilm. Co-cultures were incubated for four hours at 37 °C anaerobically to allow for bacterial integration into the streptococcal biofilms. Following integration, wells were washed twice with PBS, and biofilms were exposed to 1% (*v*/*v*) E-liquids ± flavors or 5% hydrogen peroxide (Px control) in 50% TSBY and incubated anaerobically at 37 °C for an additional 24 h.

To quantify total biofilm biomass, biofilms were washed three times with PBS, stained with 100 µL of 5% crystal violet for 10 min, and rinsed up to seven times with deionized water. Next, 100 µL of 3% acetic acid was added to all wells, and the plates were shaken at 400 rpm for 1 min to ensure complete extraction of crystal violet from the bacteria. Acetic acid solution with any crystal violet was passed to a new and clear 96-well plate. Absorbance was measured at 595 nm using the plate reader and Gen5 software (see Section 2.6), and readings were used as an index of total biofilm biomass.

#### 2.7.2. qPCR

Salivary pellicles were formed by coating acetone-treated sterile 12-well plates with 2 mL of processed human saliva per well and incubating overnight at 4 °C. *S. gordonii*, *S. intermedius*, *S. mitis*, and *S. oralis* multispecies biofilms were established, as described above (see Section 2.7.1), with a final volume of 2 mL TSBY per well, anaerobically for 24 h. *P. gingivalis* and *F. nucleatum* were grown, mixed, and integrated into streptococcal biofilms in a final volume of 2 mL, as described above (see Section 2.7.1). Following integration, biofilms were grown with 1% (*v*/*v*) E-liquids ± flavors or 5% Px or control for 24, as indicated above (see Section 2.7.1).

To quantify total bacterial DNA and *P. gingivalis* W83 DNA, biofilm DNA was collected using the DNeasy Blood & Tissue Kit from QIAGEN (REF: 69506), following the manufacturer’s instructions. The isolated DNA was stored in microcentrifuge tubes at −20 °C. TaqMan assays at 20× for *16S* with the VIC fluorescent reporter and *PG0717* with the FAM fluorescent reporter, along with the fast 2X master mix, were used for quantitative Polymerase Chain Reaction (qPCR). The StepOnePlus Real-Time PCR machine (Applied Biosystems, Foster City, CA, USA) was used to run 50 cycles, with each cycle spending 5 s at 95 °C and 20 s at 60 °C. Ct values were analyzed using the 2^−ΔCt^ method.

#### 2.7.3. Biofilm Sonication for Bacterial Dispersal and Subsequent CFU Counting

Multispecies biofilms were grown and treated with E-liquids as described above (see Section 2.7.2). Following treatments, media were removed, and the wells were washed twice with sterile PBS. Then, 3 mL PBS was added to the biofilms in the wells. Bacteria were dissociated from biofilms via probe sonication (Hielscher Ultrasonics USA, Inc. West Milford, NJ, USA) while keeping the plates on ice. Prior to each sonication session, the probe was cleaned with ethanol. The probe was submerged in PBS in each well without contacting the plate surface, and the samples were sonicated at 80% amplitude for three 10 s pulses separated by 15 s rest intervals. The resulting single-cell suspensions were serially diluted 1:10 up to 1:10^4^ in PBS, and 10 µL from each dilution was spot-plated in triplicate. For commensal streptococci, suspensions were plated onto BHI agar, then incubated aerobically at 37 °C in 5% CO_2_ for 24 h. For *P. gingivalis*, suspensions were plated onto blood agar and incubated anaerobically at 37 °C for 48 h prior to colony counting.

### 2.8. Statistical Analysis

All data is reported as means ± standard errors of the means (SEMs). All comparisons within line graphs (planktonic growth curves) and bar graphs (CFU, crystal violet and qPCR assays) were made using two-way ANOVA and one-way ANOVA, respectively, followed by Bonferroni’s multiple comparisons test. All absorbance values are normalized against background noise. CFU counts are presented in the log scale or as percentages of control. Ct values were analyzed using the 2^−ΔCt^ method. Statistical significance is indicated when *p* < 0.05. Version 5 of Prism (GraphPad Software, San Diego, CA, USA) was used to generate all graphs and perform all statistical tests.

## 3. Results

### 3.1. Effect of E-Liquid Components on P. gingivalis Planktonic Growth

**Figure 1:**
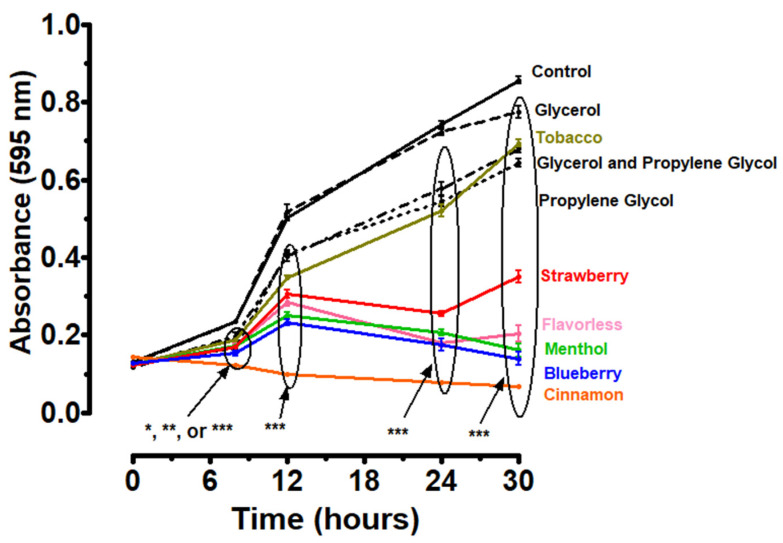
Thirty-hour growth curves illustrating the effects of E-liquids or their humectants on *P. gingivalis* planktonic growth in TSBY. Each point represents the mean ± SEM; n = 7. Two-way ANOVA with Bonferroni post hoc analysis was used to determine significance. * = *p* < 0.05, ** = *p* < 0.01 and *** = *p* < 0.001 as compared to *P. gingivalis* (control). The bubbles and arrows indicate the presence of significance.

*P. gingivalis* planktonic growth at 8 h was significantly inhibited when exposed to 1% E-liquids ± flavors (Figure 1 and Table S1). The E-liquid humectant propylene glycol and the mixture of glycerol and propylene glycol also demonstrate a similar significant inhibitory effect, from 6 h until the end of the experiment, but to a lesser extent. Glycerol shows a modest but significant inhibition only at 30 h (Figure 1 and Table S1). These results suggest that E-liquid humectants and flavors inhibit the planktonic growth of *P. gingivalis*, with flavoring agents having the most drastic effects.

### 3.2. Effects of E-Liquids and Individual Commensal Supernatants on P. gingivalis Planktonic Growth

**Figure 2:**
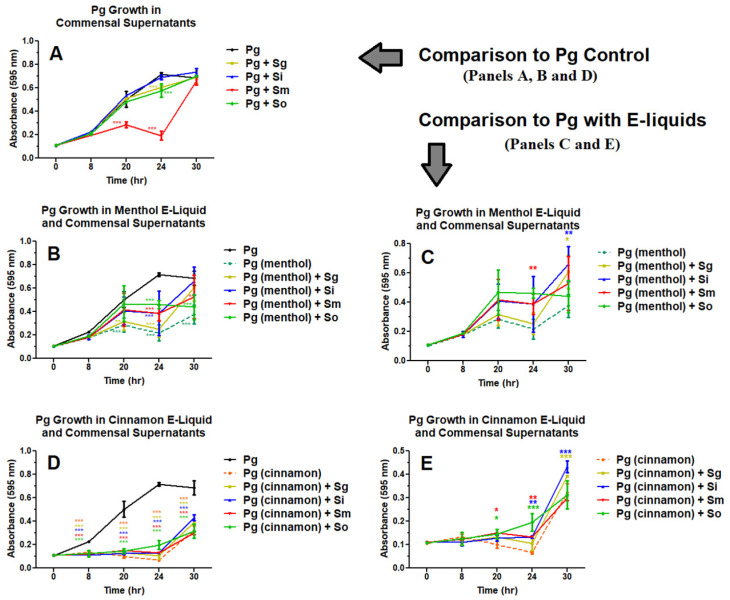
Thirty-hour growth curves illustrating the effects of individual commensal supernatants and E-liquid treatments on *P. gingivalis* (Pg) growth. Each point represents the mean ± SEM; n = 4–8. Two-way ANOVA with Bonferroni post hoc analysis was used to determine significance. * = *p* < 0.05, ** = *p* < 0.01, and *** = *p* < 0.001 as compared to *P. gingivalis* control (**A**,**B**,**D**) or as compared to *P. gingivalis* + menthol (**C**) or *P. gingivalis* + cinnamon (**E**) E-liquids. Supernatants of *S. gordonii*, *S. intermedius*, *S. mitis*, and *S. oralis* are indicated by Sg, Si, Sm, and So, respectively. Colors of the asterisks correspond to significance for Sg (yellow), Si (blue), Sm (red) and So (green) supernatants. (**C**,**E**) are enhanced views of (**B**,**D**), respectively, and indicate comparisons between E-liquids alone and E-liquids with commensal supernatants.

Our previous studies showed consistent inhibition and even cell death of oral bacteria when exposed to 3% and 5% cinnamon or menthol flavors, but no inhibition was observed at 1% [69,70,71]. Therefore, we tested the effects of 1% cinnamon and menthol flavors on *P. gingivalis* planktonic growth while exposed to individual oral commensal streptococcal supernatants (Figure 2). The growth of *P. gingivalis* is differentially influenced by the supernatants of oral commensal streptococci, especially with *S. mitis* supernatant (Figure 2A and Table S2). Other supernatants only display a modest effect, if any (Figure 2A and Table S2). As expected from Figure 1, both 1% cinnamon and menthol E-liquids severely inhibit *P. gingivalis* planktonic growth (Figure 2B,D and Table S2). Notably, when *P. gingivalis* is grown in the presence of both commensal supernatants and E-liquids, growth is slightly higher compared to E-liquid treatments alone (Figure 2C,E and Table S2). Supernatants from *S. gordonii*, *S. intermedius*, and *S. mitis*, but not *S. oralis*, modestly increase *P. gingivalis* growth in the presence of menthol-flavored E-liquid only between 24 and 30 h. In addition, all four commensal supernatants slightly increase *P. gingivalis* growth in the presence of cinnamon-flavored E-liquid between 20 and 30 h. Overall, commensal supernatants appear to limit the suppressive effects of the E-liquids, with a slightly greater effect associated with the menthol E-liquid (Figure 2 and Table S2). These results suggest that unknown metabolites in the supernatants from oral commensals can partially mitigate the toxicity of flavored E-liquids per se, thereby modulating *P. gingivalis* proliferation.

### 3.3. Effects of E-Liquids and Mixed Commensal Supernatants on P. gingivalis Planktonic Growth

**Figure 3:**
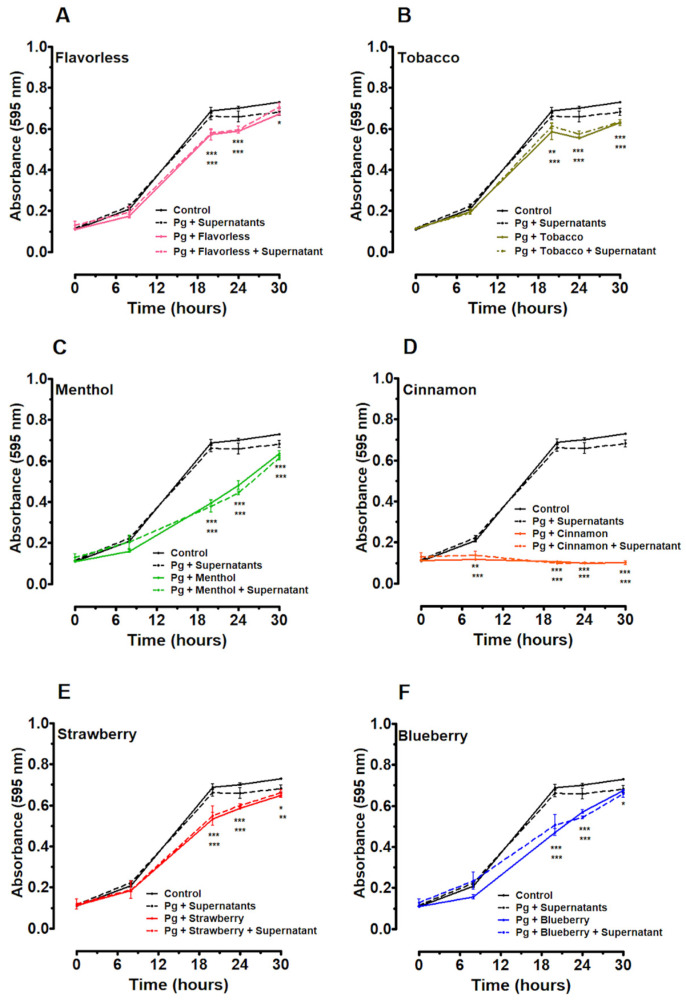
Thirty-hour growth curves illustrating the effects of mixed commensal supernatants and/or E-liquid (flavorless, tobacco, menthol, cinnamon, strawberry, and blueberry) treatments on *P. gingivalis* (Pg) growth. Supernatants are a 1:1:1:1 mixture of all four commensal supernatants. Each point represents the mean ± SEM; n = 4–6. Two-way ANOVA with Bonferroni post hoc analysis was used to determine significance. * = *p* < 0.05, ** = *p* < 0.01 and *** = *p* < 0.001 as compared to *P. gingivalis* control.

The growth of *P. gingivalis* was monitored for 30 h under conditions of exposure to E-liquids with and without mixed commensal supernatants. As expected, all E-liquids tested delayed planktonic growth kinetics significantly compared to the control, with the most significant inhibitions observed with cinnamon and menthol flavors (Figure 3 and Table S3). In the presence of mixed commensal supernatants, *P. gingivalis* exhibited growth rates similar to the control. However, whereas *P. gingivalis* growth was significantly delayed by all E-liquids (*p* < 0.001) between 20 and 24 h of growth, mixed commensal supernatants did not improve growth kinetics at any time point tested (Figure 3 and Table S3). The results of this experiment indicate that all E-liquids delay the growth of *P. gingivalis*, as can be seen in Figure 1, and that mixing the four supernatants together dampens the effects that each individual supernatant has on E-liquid inhibition (Figure 2).

### 3.4. CFU Counts of P. gingivalis at 24 h of Planktonic Growth with E-Liquids and Mixed Supernatants

**Figure 4:**
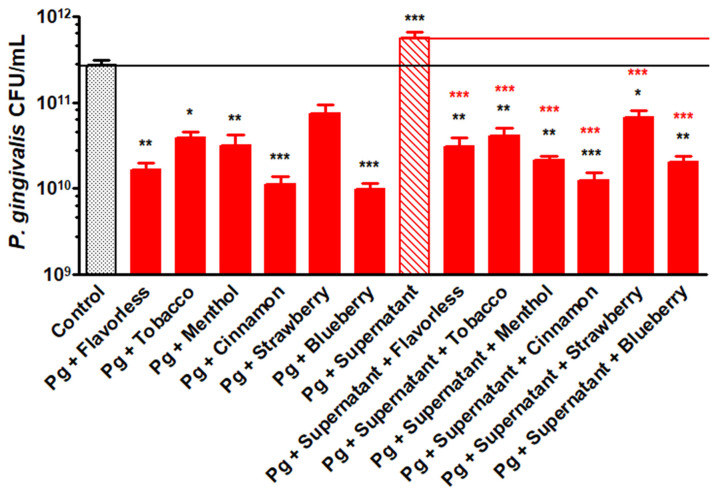
*P. gingivalis* CFUs/mL at 24 h of planktonic growth. Each value represents the mean ± SEM of CFUs, n = 8 to 12. One-way ANOVA with Bonferroni post hoc analysis was used to determine significance. * = *p* < 0.05, ** = *p* < 0.01 and *** = *p* < 0.001 as compared to *P. gingivalis* (Pg) control. *** = *p* < 0.001 as compared to Pg + supernatant. The black and red horizontal lines indicate levels of Pg control and Pg + supernatant, respectively.

Figure 4 shows *P. gingivalis* CFUs/mL at the 24 h time point of planktonic growth shown in Figure 3. The *P. gingivalis* control yields 2.7 × 10^11^ CFUs/mL, but when exposed to 10% mixed commensal supernatants, *P. gingivalis* CFU/mL nearly doubles to 5.6 × 10^11^. However, when exposed to E-liquids, CFU counts significantly decrease by approximately an order of magnitude, except for when exposed to strawberry E-liquid (Figure 4 and Table S4). *P. gingivalis* CFU counts in the presence of mixed supernatants from all four commensals and E-liquids also show a significant decrease compared to the control and the control with mixed supernatants. CFU counts are not significantly different between treatments with E-liquids alone as compared to their respective E-liquids plus supernatants. These results indicate that all E-liquids significantly decrease the planktonic growth of *P. gingivalis* and that the mixed commensal supernatants do not alter these effects.

### 3.5. Quantification of E-Liquid Effects on Multispecies Biofilm Biomass via Crystal Violet Assay

**Figure 5:**
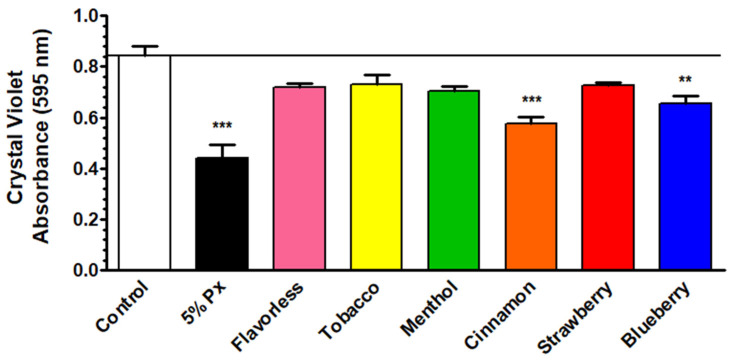
Crystal violet assay of multispecies biofilms. Multispecies biofilm biomass after treatments with 1% E-liquids ± flavors in 50% TSBY. Each bar represents the mean ± SEM of absorbance (n = 10 to 12). One-way ANOVA with Bonferroni post hoc analysis was used to determine significance. ** = *p* < 0.01 and *** = *p* < 0.001 as compared to control. The black horizontal line indicates the level of control of biofilms.

The crystal violet assay was employed to test the effects of E-liquids on the total biofilm biomass of oral multispecies biofilms, including the four Gram-positive commensals and the two Gram-negative anaerobes. Figure 5 (Table S5) displays that biofilm biomass of oral bacteria is significantly affected by 1% E-liquids with cinnamon and blueberry compared to the control, but not with the other flavor treatments. The hydrogen peroxide treatment was used as a control and caused an expected significant reduction in biofilm biomass. These results indicate that at least some of the E-liquids tested reduce total biofilm biomass.

### 3.6. Quantification of E-Liquid Effects on Multispecies Biofilm Biomass via qPCR

**Figure 6:**
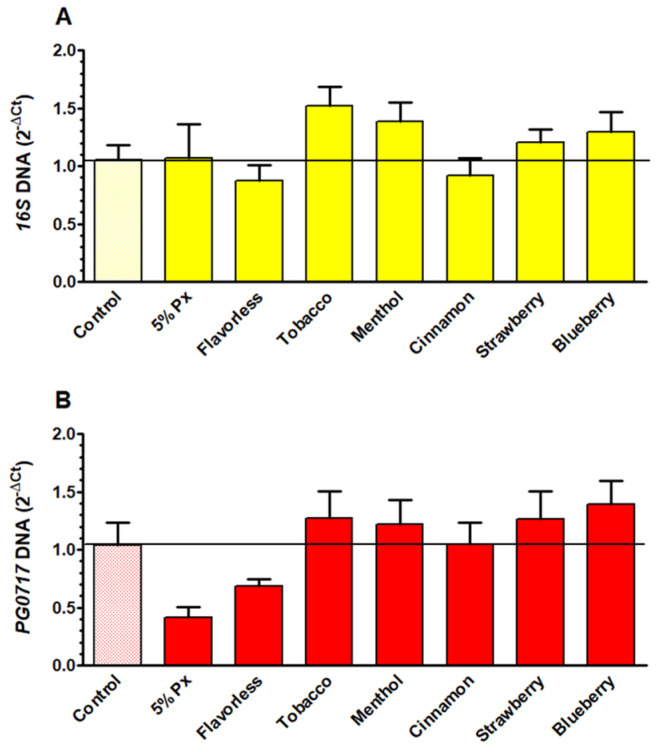
Quantitative PCR results comparing total bacterial DNA (**A**) and *P. gingivalis* DNA (**B**) from multispecies biofilms after treatment with 1% E-liquids ± flavorings in 50% TSBY. Each bar represents the mean ± SEM of 2^−ΔCt^ (n = 8 to 16). One-way ANOVA with Bonferroni post hoc analysis was used to determine significance. The black horizontal lines indicate the level of control biofilms.

To test the effects of E-liquids on total bacterial DNA and *P. gingivalis* DNA within the multispecies biofilms, a qPCR assay was performed. Total bacterial DNA (Figure 6A and Table S6) and *P. gingivalis* DNA (Figure 6B and Table S6) quantifications yielded no significant differences between treatment groups and their respective controls. This data indicates that levels of both bacterial and *P. gingivalis* DNA are not significantly altered following E-liquid treatments on biofilms. This could be due to DNA quantification accounting for both live and dead bacteria as well as DNA found in the slime layer surrounding biofilms.

### 3.7. Quantification of E-Liquid Effects on Multispecies Biofilm Biomass via CFU Counting

**Figure 7:**
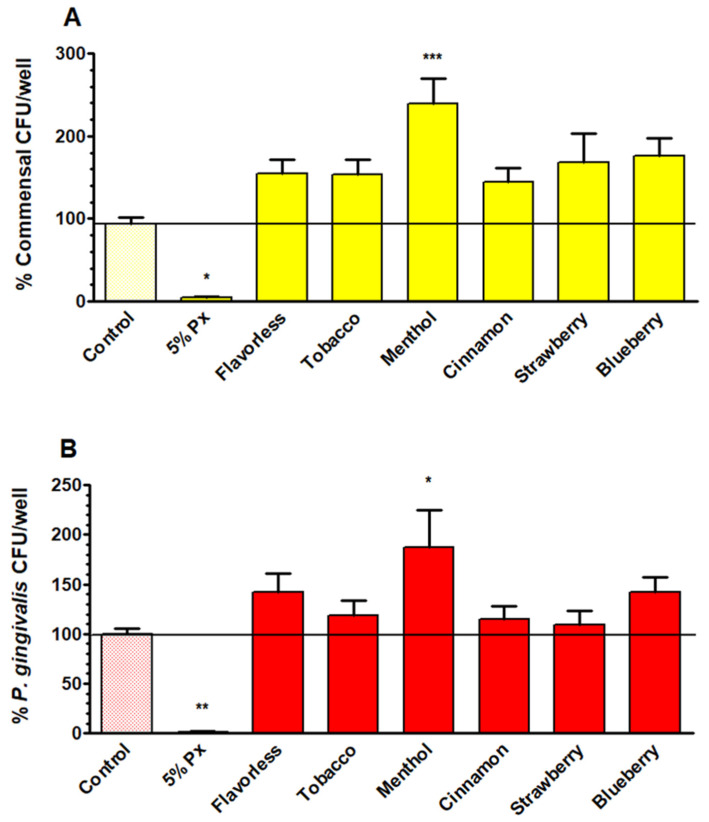
CFU counts comparing commensal bacteria (**A**) and *P. gingivalis* (**B**) from multispecies biofilms after treatment with 1% E-liquids ± flavorings in 50% TSBY. Each bar represents the mean ± SEM of CFUs/well (n = 13 to 18). One-way ANOVA with Bonferroni post hoc analysis was used to determine significance. * = *p* < 0.05, ** = *p* < 0.01, and *** = *p* < 0.001 as compared to commensal bacteria or *P. gingivalis* controls. The black horizontal lines indicate the level of control biofilms.

To test the effects of E-liquids on viable commensals and *P. gingivalis* within the multispecies biofilms following 1% E-liquid treatments, a CFU viability test was performed. Across all E-liquid conditions, both commensals (Figure 7A and Table S7) and *P. gingivalis* (Figure 7B and Table S7) showed increased CFU counts compared with controls, but only the treatment with menthol E-liquid significantly increased viable counts. The results indicate that *P. gingivalis* and commensal bacteria CFUs are slightly higher in biofilms when exposed to E-liquids.

### 3.8. Comparison of E-Liquid Effects on P. gingivalis Growth Planktonically and in Multispecies Biofilms

To compare the effects of E-liquids on *P. gingivalis* growth, both planktonically and in multispecies biofilms, the CFUs from Figure 4 and Figure 7B are compared side by side and expressed as percents of control in Table 1. Under planktonic conditions, *P. gingivalis* growth was severely decreased, with percent values ranging between 4% and 27% after exposure to 1% E-liquids. On the other hand, in multispecies biofilms exposed to 1% E-liquids, the relative percentages of *P. gingivalis* CFUs were similar and in some cases higher than those of the control. For example, menthol E-liquid treatment induced an 87% increase with respect to the control, while flavorless and blueberry E-liquids yielded a 42% increase. Taken altogether, these results indicate that E-liquids favor *P. gingivalis* growth in oral multispecies biofilms.

## 4. Discussion

Homeostasis in the oral cavity is maintained by interactions between oral microbes and the host [75,76]. *P. gingivalis* often enters as an opportunistic invader [77,78,79]. Studying *P. gingivalis* in multispecies biofilms, such as the species community in this study, offers a more realistic model of oral microbiology. Our results show that E-liquids are detrimental to *P. gingivalis* when isolated and grown planktonically. However, when *P. gingivalis* is exposed to E-liquids as part of a polymicrobial community its growth is not inhibited. It is unlikely that nicotine within the E-liquid contributes to this detrimental effect. The nicotine concentration used in the current study was 200 µg/mL, and Huang et al. (2014) demonstrated that nicotine has no effects on *S. gordonii* at concentrations lower than 1 mg/mL [80]. Furthermore, it must be noted that the amount of E-liquid used in this in vitro study is approximately 14 times greater than the amount of aerosolized E-liquid inhaled as 100 puffs, assuming that 20% of the aerosol is integrated into the saliva before it is diluted and/or swallowed (unpublished data).

When *P. gingivalis* is grown planktonically, exposure to E-liquids ± flavors induces a dramatic decrease in growth and CFU production (Figure 1 and Figure 4), but in the presence of commensal supernatants, the toxicity of the flavoring agents is mitigated, thereby promoting *P. gingivalis* growth (Figure 2). These results indicate that commensal bacteria may provide protective mediators or metabolites that partially counteract the toxic effects of E-liquids. It is for this reason that the effects of E-liquids on *P. gingivalis* were tested in the context of multispecies biofilms. Within these microbial communities, commensal bacteria may alter the microenvironment by providing metabolites that neutralize the E-liquid toxicity. Furthermore, because of the anaerobic nature of *P. gingivalis*, this species typically tends to live in the deeper layers of oral biofilms, away from toxic agents, such as E-liquids and their components, which dilute to sub-lethal concentrations as they approach the *P. gingivalis* microenvironment.

Figure 1 suggests that E-liquid components inhibit *P. gingivalis* growth, particularly those of flavored E-liquids such as cinnamon, blueberry, and menthol E-liquids. Meanwhile, E-liquid humectants, propylene glycol and glycerol, affect *P. gingivalis* growth to a lesser extent, suggesting that the flavoring agents are the most disruptive to *P. gingivalis* growth. The present investigation aligns with other studies suggesting that flavored E-liquids inhibit the growth of oral bacteria similarly to aerosols [41,69,70,71,81].

Figure 2A demonstrates that commensal supernatants, especially those of *S. mitis*, decrease the growth of *P. gingivalis*, which agrees with the results obtained by others. For example, *S. mitis* produces an abundant amount of hydrogen peroxide in comparison to the other commensals, inhibiting the biofilm formation of the cariogenic species *Streptococcus mutans* [82]. In addition to hydrogen peroxide, commensal bacteria play a protective role in maintaining microbial balance by producing competitive scavenging metabolites, reactive nitrogen intermediates, and bacteriocins, all of which help protect against pathogenic species [83]. However, supernatants from commensal species seem to partially enhance *P. gingivalis* planktonic growth when exposed to menthol and cinnamon E-liquids (Figure 2C,E). Previous studies from our group have shown that cinnamon and menthol flavors have the greatest inhibitory impact on the growth of oral bacteria [69,70,71]. This study displays the same trend with *P. gingivalis*, as can be seen in Figure 1, and hence these two flavors were used to test the effects of individual commensal supernatants in Figure 2. Molecular approaches indicate that the ecological balance in the oral cavity is maintained through antagonistic and mutualistic interactions among species [51,84,85,86]. Such interactions are mediated by metabolites [51,60,76,85,86,87] that could also participate in the tolerance to external toxic materials [88], such as E-liquids. The materials responsible for such beneficial effects remain unknown and should be investigated.

The protective effects noticed by the individual commensal supernatants (Figure 2) are lost when mixed at a 1:1:1:1 ratio (Figure 3 and Figure 4). Likely, such protective materials in the supernatants either fall below the threshold of activity when diluted 1:4 or unknown biochemical interactions change their functions. Although this study tested a small number of flavors compared to the plethora of flavors available [89,90], the flavors tested are well-represented in the scientific literature [91] and help us understand the tolerance of *P. gingivalis* to these external agents.

Six species were chosen to grow in vitro oral biofilms and test the viability of *P. gingivalis* in this context. In addition to all four commensals, *F. nucleatum* was included because it is required as a bridging species between *P. gingivalis* and the four commensal streptococci [52]. Most flavors, except cinnamon and blueberry, do not significantly alter biofilm biomass (Figure 5). Despite the reductions in the cinnamon and blueberry treatments, the overall magnitude of biomass reduction across treatments was minimal. These findings extend prior research showing that vaping induces variable effects on oral bacteria, including commensal streptococci, depending on the dose, physical state (liquid vs aerosol) and flavors [69,70,71,81]. The reduced biomass may be attributable to the E-liquid components or their flavorings. For example, trans-cinnamaldehyde, a component of the cinnamon flavor, can inhibit biofilm formation of *S. mutans* [92]. Crystal violet staining quantifies total biomass, making it difficult to determine the exact biofilm composition.

Biofilms exposed to E-liquids showed minimal differences relative to the control in terms of both *16S* (total bacteria) and *PG0717* (*P. gingivalis* only) DNA (Figure 6). These results indicate that the biofilm microenvironment offers substantial protection to *P. gingivalis* from E-liquids. While individual commensal supernatants partially protect *P. gingivalis* against E-liquids (Figure 2), biofilm communities offer a greater degree of protection, not only to *P. gingivalis* but to the commensals as well. Furthermore, E-liquid treatments yielded a modest increase in CFU counts from multispecies biofilms for both commensal streptococci (Figure 7A) and *P. gingivalis* (Figure 7B) relative to the untreated control. In contrast to planktonic results (Figure 1, Figure 2, Figure 3 and Figure 4), as well as previous studies reporting antimicrobial/antibiofilm effects of flavored E-liquids on oral bacteria and streptococcal communities [69,70], the data suggests that biofilms mitigate E-liquid toxicity compared to planktonic growth, consequently yielding higher CFU counts. Even though total biofilm biomass (Figure 5) and DNA levels (Figure 6) show minimal changes after E-liquid exposure, it is likely that multispecies biofilms may specifically increase the number of CFUs (Figure 7) rather than increasing overall biofilm mass, slime layer, dead cells, or total DNA. In other words, E-liquids may increase CFUs within the biofilms (i.e., live material) and not the slime layer or other non-living materials, without producing a substantial change in crystal violet staining or DNA readouts. Notably, menthol stands out as the only treatment causing a significant CFU increase for both commensals and *P. gingivalis* (Figure 7). This provides a basis for understanding why polymicrobial interactions in a multispecies biofilm could alter responses to the same stressor. This supports the broader idea that polymicrobial interactions and metabolite exchange within oral biofilms can change how stressors affect individual species like *P. gingivalis* [83,87,93]. Despite the established bactericidal properties of E-liquids [71], *P. gingivalis* growth is not inhibited in the context of polymicrobial oral biofilms in vitro, and this is consistent with studies that indicate that vaping contributes to dysbiosis in oral biofilms [91]. Similarly, a study by Ganesan et al. (2020) [94] used a similar polymicrobial model but included a larger array of oral species grown in vitro and exposed to ECIG aerosols for 24 h. Ganesan and co-workers also found a shift to a more pathogenic microbial community after treatments [94]. Nonetheless, this study only included six species of oral bacteria and a single 24 h exposure to E-liquids and did not take into account the host response, which are limitations to consider when extrapolating these findings to in vivo scenarios.

Our findings demonstrate that while E-liquid flavoring agents are directly toxic to *P. gingivalis* in planktonic culture, individual commensal supernatants modestly improved growth of this anaerobe. Table 1 illustrates the striking contrast between planktonic and biofilm growth: while E-liquids reduce *P. gingivalis* viability during planktonic growth, multispecies biofilm conditions abolish this effect, with *P. gingivalis* CFUs either at control levels or higher across all tested flavors. Therefore, when multispecies oral biofilms are exposed to E-liquids, *P. gingivalis* grows well. This correlates with clinical observations reporting that vapers exhibit elevated *P. gingivalis* levels and face an increased risk of periodontal disease compared to non-vapers [36,91]. Vapers exhibit a unique periodontal microbiome characterized by an increase in pathobionts, alongside increased inflammatory markers, and other clinical signs of periodontitis [41]. The *P. gingivalis* colonization observed in vapers may have serious systemic implications, as the pathobiont has been linked to cardiovascular disease, arthritis risk, and diabetes [44,45].

Our results compare favorably with results from the clinical literature demonstrating that vaping promotes oral dysbiosis and elevates periodontal pathogens. Clinically, vapers often show depletion of commensal species, while pathobionts are increased, and metabolomic analyses identify vaping-induced metabolites that directly enhance oral cancer progression [95]. Such metabolic findings suggest that pathogenic bacteria like *P. gingivalis* are protected and thrive in biofilm microenvironments when exposed to vaping. Our findings contrast with clinical reports in that both commensals and *P. gingivalis* show elevated CFU counts after E-liquid exposure (Figure 7), whereas clinically commensals are depleted [33,34]. This discrepancy may be attributed to the limited microbiome in our model, as well as the absence of the host immune response. Furthermore, in our model, E-liquid-treated media were used as opposed to saliva exposed to aerosols. Simply put, heating and aerosolization of E-liquids contribute unknown factors that alter the oral microbiome which are not easily replicated in vitro. However, the overarching conclusion of the broader literature is that aerosols favor the growth of pathobionts, which is consistent with our results.

## 5. Conclusions

In conclusion, this study demonstrates that when *P. gingivalis* is exposed to E-liquids planktonically, its growth is inhibited, but when grown in multispecies biofilms, this inhibition is abolished. This study supports the paradigm that E-liquids alter microbial composition and provides preliminary in vitro evidence consistent with clinical reports (in vivo). E-liquids disturb oral biofilms, pushing towards *P. gingivalis* dysbiosis. The interactions between *P. gingivalis* and other oral bacteria in the context of biofilms exposed to E-liquids require further study regarding their relevance to clinical scenarios associated with vaping and the risks for periodontal disease.

## Figures and Tables

**Table 1:** Comparison of *P. gingivalis* CFUs after planktonic growth and multispecies biofilm growth during exposure to 1% E-liquids and flavors.

	Control	Flavorless	Tobacco	Menthol	Cinnamon	Strawberry
Planktonic *	100	6	14	11	4	27
Biofilm **	100	142	119	187	115	109

* Data obtained from Figure 4. ** Data obtained from Figure 7B. All data is presented as percentages from controls.

## Data Availability

The original contributions presented in this study are included in the article/Supplementary Material. Further inquiries can be directed to the corresponding author.

## Supplementary Materials

The following supporting information can be downloaded at https://www.mdpi.com/article/10.3390/dj14030172/s1: Table S1: Effect of E-Liquid Components on *P. gingivalis* Planktonic Growth; Table S2: Effects of E- >Liquids and Individual Commensal Supernatants on *P. gingivalis* Planktonic Growth; Table S3: Effects of E-Liquids and >Mixed Commensal Supernatants on *P. gingivalis* Planktonic Growth; Table S4: CFU Counts of *P. gingivalis* at 24 h of >Planktonic Growth with E-Liquids and Mixed Supernatants; Table S5: Quantification of E-Liquid Effects on Multispecies Biofilm Biomass via Crystal Violet Assay; Table S6: Quantification of E-Liquid Effects on Multispecies Biofilm Biomass via qPCR; Table S7: Quantification of E-Liquid Effects on Multispecies Biofilm Biomass via CFU Counting.
